# Sn‐Based Perovskite for Highly Sensitive Photodetectors

**DOI:** 10.1002/advs.201900751

**Published:** 2019-07-13

**Authors:** Chun‐Ki Liu, Qidong Tai, Naixiang Wang, Guanqi Tang, Hok‐Leung Loi, Feng Yan

**Affiliations:** ^1^ Department of Applied Physics The Hong Kong Polytechnic University Hung Hom Kowloon Hong Kong

**Keywords:** flexible electronics, gain, lead‐free perovskites, photodetectors, responsivity

## Abstract

Organic–inorganic hybrid perovskites have emerged as promising functional materials for high‐performance photodetectors. However, the toxicity of Pb and the lack of internal gain mechanism in typical perovskites significantly hinder their practical applications. Herein, a low‐voltage and high‐performance photodetector based on a single layer of lead‐free Sn‐based perovskite film is reported. The device shows broadband response from ultraviolet to near‐infrared light with a responsivity up to 10^5^ A W^−1^ and a high gain at a low operating voltage. The outstanding performance is attributed to the high hole mobility, p‐doping nature, and excellent optoelectronic properties of the Sn‐based perovskite. Moreover, the device is assembled on a flexible substrate and demonstrates both high sensitivity and good bending stability. This work demonstrates a route for realizing nontoxic, low‐cost, and high‐performance perovskite photodetectors with a simple device structure.

Photodetectors (PDs) have been widely used for various applications, such as optical communication, biomedical imaging, health care monitoring, video imaging, building inspection, and night vision.^1^, ^2^, ^3^ However, commercial III–V compound or Si‐based PDs normally suffer from relatively low response, high driving voltage, limited spectral response, and expensive fabrication.^4^, ^5^, ^6^, ^7^ Thus, it is necessary to develop the next‐generation PDs based on novel materials and designs. Recently, organic–inorganic halide perovskites (OIHPs) have gained much research interests for their applications in optoelectronic devices, including solar cells, PDs, and light emission diodes (LEDs), owing to their superior physical properties, including high light absorption coefficient, direct bandgap, low exciton binding energy, and long carrier lifetimes.^8^, ^9^, ^10^, ^11^, ^12^, ^13^, ^14^ OIHPs have a structure of ABX_3_, where A site is a monovalent cation like methylammonium (MA), formamidinium (FA), or cesium (Cs), B is a divalent metal cation such as lead (Pb) or tin (Sn), and X is a halide anion, such as chlorine (Cl), iodine, or bromine.^15^ Notably, thin perovskite films could be conveniently fabricated on both rigid and flexible substrates through a low‐temperature solution process, which are particularly suitable for applications in some emerging fields like flexible and wearable electronics.^16^, ^17^, ^18^

So far, most research efforts on OIHP‐based PDs have been focusing on Pb‐containing perovskites mainly due to their success in photovoltaic cells.^13^, ^19^, ^20^, ^21^ For instance, a planar flexible MAPbI_3_ photoconductor with a responsivity of 3.49 A W^−1^ was reported by Hu et al.^19^ Deng et al. reported a PD based on single‐crystalline MAPbI_3_ microwires, which showed a responsivity of 13.5 A W^−1^.^20^ An ambipolar phototransistor having responsivity up to 320 A W^−1^ was developed by Li at el.^13^ The relatively limited responsivity could be attributed to the lack of additional gain mechanism and the unsatisfactory charge transport properties of the perovskite film.^22^ Although much higher responsivity and gain can be achieved in PDs by introducing heterostructures in the devices,^22^, ^23^, ^24^, ^25^ the additional processing steps and materials involved in the devices may complicate the fabrication procedure and increase the cost. More importantly, the applications of the aforementioned perovskite PDs can be hindered by the toxicity of Pb.^26^ In light of this, some works have been done on lead‐free perovskite‐based PDs, yet satisfactory results are still lacking.^27^, ^28^, ^29^, ^30^, ^31^ For instance, Waleed et al. fabricated a MASnI_3_ nanowire‐array PD by using a vapor phase chemical reaction method with porous alumina template, exhibiting a responsivity and detectivity of only 0.47 A W^−1^ and 8.80 × 10^10^ Jones, respectively.^27^ A red‐light PD based on CsBi_3_I_10_ was developed by Tong et al., which demonstrated a low responsivity of only 21.8 A W^−1^.^28^ Qian et al. developed a flexible PD based on (PEA)_2_SnI_4_, a 2D perovskite, with a limited responsivity of 16 A W^−1^.^29^ So, the lead‐free perovskite PDs demonstrated relatively low responsivity and gain.

In this paper, we report a high‐performance PD based on a single layer of lead‐free perovskite material FASnI_3_. Pristine FASnI_3_ is unstable in air because Sn^2+^ can be easily oxidized to Sn^4+^. By introducing hydroxybenzene sulfonic acid additive along with excess SnCl_2_ to encapsulate perovskite grains, air‐stable FASnI_3_ films were successfully prepared as the PDs.^32^ The device based on the stable FASnI_3_ layer displays high responsivity in a broad wavelength region from 300 to 1000 nm with the maximum responsivity and gain over 10^5^ A W^−1^ and 10^5^, respectively, at a low working voltage. The intrinsic high gain in the photosensitive material can be attributed to the high hole carrier mobility and photocarrier density in the FASnI_3_ perovskite owing to its p‐doping nature. Moreover, the PD is successfully fabricated on flexible substrate and demonstrates both high sensitivity and robust bending stability. The results suggest that the lead‐free FASnI_3_ perovskite is a promising material for high‐performance PDs.

**Figure** **1**a displays the schematic diagram of a PD based on a pure FASnI_3_ perovskite film. Cr/Au electrodes are patterned on n+ Si/SiO_2_ substrates by photolithography. A 120 nm thick FASnI_3_ film is then coated on the channel region with a solution process. In the preparation of the FASnI_3_ film, a reducing agent hydroquinone sulfonic acid (KHQSA) is added in the precursor solution to improve the film stability in air.^32^ The sulfonate group in KHQSA interacts with Sn^2+^ ion to form a SnCl_2_–KHQSA complex layer and induce in situ encapsulation of perovskite grains. This antioxidant layer can greatly enhance the air stability of the perovskite film. Under light illumination, electron–hole (e–h) pairs and excitons are generated in the perovskite film. Due to the low exciton binding energy of the perovskite, excitons upon generation would dissociate into e–h pairs rapidly.^33^ Under an external bias, photocarriers are then collected by the electrodes at two ends, leading a photoresponse of the device.

**Figure 1:**
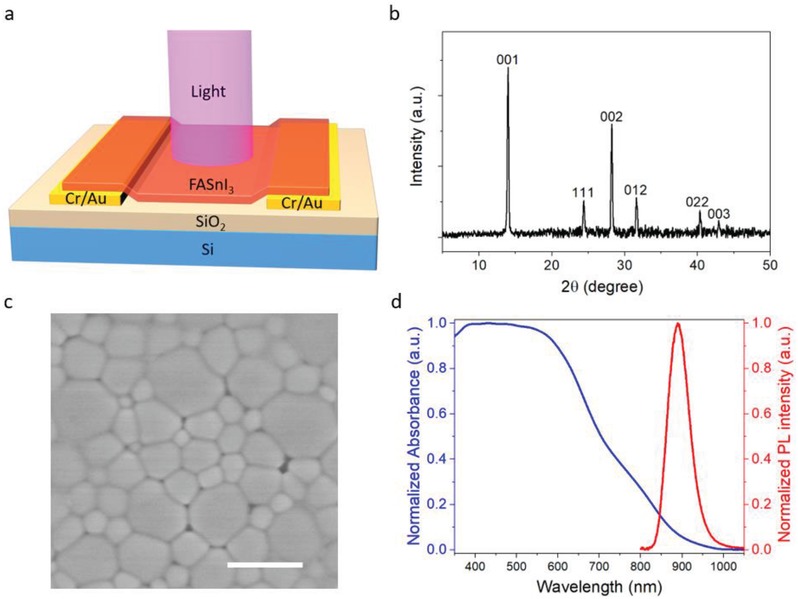
Illustration of device design and material characterization for the FASnI_3_ PD. a) Schematic diagram of the PD. b) XRD pattern of the FASnI_3_ film on glass substrate. c) SEM image for the perovskite film on SiO_2_/Si substrate, where the scale bar is 1 µm. d) Photoluminescence and absorption spectrum of the perovskite film on glass substrate.

The crystallinity and structure of the FASnI_3_ perovskite film coated on a glass substrate was characterized by X‐ray diffraction (XRD), as shown in Figure 1b. All peaks in the XRD pattern are corresponding to the α‐phase with a orthorhombic structure, with space group of Amm2 and lattice parameters of *a* = 6.3286 Å, *b* = 8.9554 Å, and *c* = 8.9463 Å, indicating that the perovskite film has a single phase of the expected structure.^34^ The sharp and intensive (001) and (002) peaks also indicate a highly oriented polycrystalline perovskite film on the substrate. The surface morphology of the film was observed under scanning electron microscopy (SEM), as shown in Figure 1c. The perovskite film has a large average grain size (≈500 nm) and can densely cover the substrate with good uniformity, which is beneficial for charge transport in the PD. Additionally, the optical bandgap was revealed by photoluminescent (PL) spectroscopy and UV–vis spectroscopy. As shown in Figure 1d, the sample displayed a sharp PL peak and an absorbance edge at around 890 nm, indicating its direct bandgap of ≈1.4 eV. The electronic properties of the film were also investigated using Hall effect measurement. As shown in Table S1 in the Supporting Information, the hole mobility and carrier concentration are estimated as ≈19 cm^2^ V^−1^ s^−1^ and ≈4.6 × 10^16^ cm^−3^, respectively. The mobility value obtained here is consistent with those reported before by optical methods.^35^, ^36^ Such high mobility further confirms the high quality of the film and is desirable for carrier transport in PDs. On the other hand, the high carrier density of the perovskite film can be attributed to the heavily p‐doping caused by partial oxidation of Sn^2+^ cation into Sn^4+^.^37^

**Figure** **2**a shows the channel current versus applied voltage (*I*–*V*) curves of the PD under light illumination with a wavelength of 685 nm at different intensities. The linear *I*–*V* relationship in the dark indicates the ohmic contact at the perovskite/Au interface, which is favorable for efficient charge collection. The photocurrent (*I*_ph_) of the device could be defined by the following equation

(1) $$I_{\mathrm{ph}}\;=\;I_{\mathrm{light}}\;-\;I_{\mathrm{dark}}$$

where *I*_light_ and *I*_dark_ are currents through device channel when light is on and off, respectively. Figure 2b depicts the *I*_ph_ versus drain voltage under light illumination at the wavelength of 685 nm. *I*_ph_ of the device increases with the increase of the light intensity as well as the drain voltage. Similar photoresponse to light illumination with different wavelengths, including 420 and 850 nm, are also obtained (see Figure S1a–d, Supporting Information).

**Figure 2:**
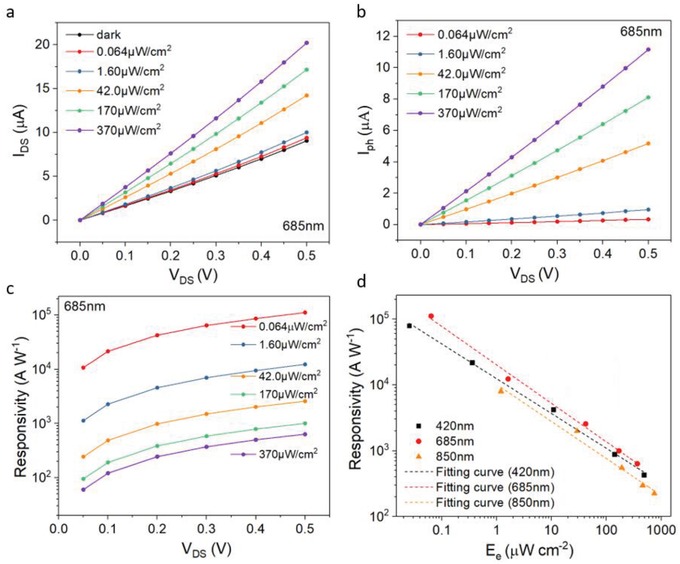
Device performance. a) *I*–*V* curves in dark and under illumination for the perovskite device. b) Photocurrent as a function of drain voltage for a FASnI_3_ device under illumination of light with 685 nm wavelength at different intensities. c) Responsivity versus drain voltage relationship under different intensities of light with 685 nm wavelength. Solid dots are experimental data. d) Responsivity versus light intensity under light illumination at wavelengths of 420, 685, and 850 nm, respectively. Applied voltage is 0.5 V and the dotted lines are fitting curves with a formula of $R\;\propto \;E_{e}^{\beta -1}$.

One important parameter for the sensitivity of a PD is the responsivity (*R*) given by

(2) $$R\;=\;\frac{I_{\mathrm{ph}}}{WLE_{e}}$$

where *W* and *L* are channel width and length, and *E*_e_ is light intensity.^38^ Figure 2c displays the relationship between responsivity and driving voltage for different intensities under 685 nm illumination. The maximum responsivities can be as high as 1.1 × 10^5^ A W^−1^ under a low driving voltage of 0.5 V. This value is several orders of magnitude higher than other perovskite‐based PDs with similar structure, i.e., PDs only consist of a single layer of perovskite film.^13^, ^19^, ^28^, ^29^ The comparison of our PDs with others is presented (see Table S2, Supporting Information). The relationship between responsivity and illuminated light intensity of the same device under 420, 685, and 850 nm light is revealed in Figure 2d. The high responsivity can be attributed to the large gain of the device. The gain (*G*) or external quantum efficiency of a PD is a measurement on how many photocarriers are generated upon per unit incident photon per unit time and given by^38^, ^39^, ^40^, ^41^

(3) $$G\;=\;\frac{I_{\mathrm{ph}}/q}{P_{\mathrm{in}}/U_{\mathrm{ph}}}\;=\;\frac{Rhc}{q\lambda }$$

where *q* is the elementary charge, *P*_in_ is optical power of incident light, *U*_ph_ is incident photon energy, *c* is the vacuum light speed, *h* is Planck's constant, and λ is the incident light wavelength. With the highest responsivity at 685 nm, the maximum gain is calculated to be as high as 2.0 × 10^5^.

To better understand the mechanism for the high performance of the FASnI_3_ PD, we also prepared a PD based on a typical lead‐based perovskite MAPbI_3_ film with a thickness of 300 nm. The device performance of the MAPbI_3_ PD was characterized systematically at the same condition for the FASnI_3_ PD (see Figure S2a–c, Supporting Information). Under light illumination at 685 nm wavelength, the maximum responsivity is 0.43 A W^−1^, which is comparable to previously reported results.^19^ The corresponding gain is only 0.78, which is five orders of magnitude lower than that of the FASnI_3_ PD. Since the carrier mobility of MAPbI_3_ is very close to that of FASnI_3_, the much higher responsivity cannot be correlated to the difference in the mobilities of the two materials.^42^ So the much higher gain in the FASnI_3_ PD indicates that a unique amplification mechanism should exist in the device, as explained in the following model. We consider that the low photoresponse of the MAPbI_3_ device is attributed to the trapping of both photogenerated electrons and holes in the perovskite layer by crystal defects and grain boundaries. On the contrary, FASnI_3_ film is highly p‐type doped, indicating that the Fermi level is close to the valence band in the perovskite film (see Figure S3, Supporting Information). Most of traps are already filled with holes and, consequently, the photogenerated holes can conduct current in the valence band. Meanwhile, photogenerated electrons can be easily trapped in the FASnI_3_ perovskite film and thus the lifetime of electrons is rather long. As a result, the photogenerated holes can circulate lots of times (equal to the gain) in the perovskite layer before recombining with the trapped electrons. This effect leads to the high gain of the FaSnI_3_ PD, which is far superior than other perovskite PDs with similar structure, as listed in Table S2 in the Supporting Information.^13^, ^19^, ^28^, ^29^ Actually, high gain PDs enabled by selective trapping of photocarriers at defect states were reported before for other materials.^43^, ^44^ However, this effect has not been discovered for PDs based on OIHPs until now. Moreover, the carrier lifetime of FASnI_3_ measured by time‐resolved photoluminescent (TRPL) is typically much shorter than that of MAPbI_3_.^32^, ^45^ This is because TRPL can only measure radiative recombination lifetime. Most of the photogenerated electrons in FASnI_3_ are trapped before they can radiatively recombine with holes. After that, nonradiative recombination (trap‐assisted) dominates. The relatively long decay time in the FASnI_3_ PD evidences the slow nonradiative recombination process in the film, which gives the device a high gain.

Notably, the responsivity of the device decreases with the increase of light intensity, following a relationship applicable to many PDs:^46^, ^47^ $R\;\propto \;E_{e}^{\beta -1}$, where β is a fitting constant and *E*_e_ is light intensity. This phenomenon is due to the increased carrier recombination when more photocarriers are generated and accumulated in the perovskite film. Assuming the hole mobility upon light illumination is unchanged, the photocarrier density can be estimated according to the following equation

(4) $$x_{\mathrm{ph}}\;=\;\frac{L}{q\mu WV_{\mathrm{DS}}}\;I_{\mathrm{ph}}$$

where *x*_ph_, *µ*, and *V*_DS_ are photocarrier density, mobility, and drain voltage, respectively.^38^ Using the mobility measured by Hall effect measurement, the photocarrier density as a function of light intensity can be thus calculated (see Figure S4, Supporting Information). Under illumination of 685 nm light with the intensity of 370 µW cm^−2^ and at a working voltage of 0.5 V, the maximum photocarrier density is calculated to be 2.6 × 10^10^ cm^−2^. The corresponding bulk density is estimated to be 2.2 × 10^15^ cm^−3^.

Specific detectivity (D*) is a figure of merit for PDs and given by^38^

(5) $$D*\;=\;\frac{{\left(AB\right)}^{1/2}}{P_{n}}$$

(6) $$P_{n}\;=\;\frac{{\bar{i_{n}^{2}}}^{1/2}}{R}$$

where *A* is active area of the PD, *B* is the bandwidth, *P*_n_ is noise equivalent power (NEP), ${\bar{i_{n}^{2}}}^{1/2}$ is the root mean square value of the noise current, and *R* is the responsivity of the device. The noise level at bandwidth of 1 Hz was found to be 0.4 nA (see Figure S7, Supporting Information),^23^ which yields a NEP of 3.6 × 10^−15^ W. D* of the PD is calculated to be 1.9 × 10^12^ Jones at an applied voltage of 0.5 V and illumination wavelength of 685 nm, which is either higher or comparable to other PDs with similar design (including the Pb‐containing ones).^13^, ^19^, ^28^, ^29^

Spectral response is another key issue for a PD. The normalized spectral responsivity and gain are displayed in **Figure** **3**a. The PD exhibits photoresponse from UV to NIR (300 to 1000 nm) with a sharp downfall beyond 850 nm, which is correlated to the absorption edge and bandgap of FASnI_3_. In the wavelength region beyond the bandgap of perovskite (≈890 nm), the device response can be attributed to the excitation of electrons from the valence band to the trap states within the bandgap with low possibility,^24^ and consequently the responsivity is much lower than that in the visible region.

**Figure 3:**
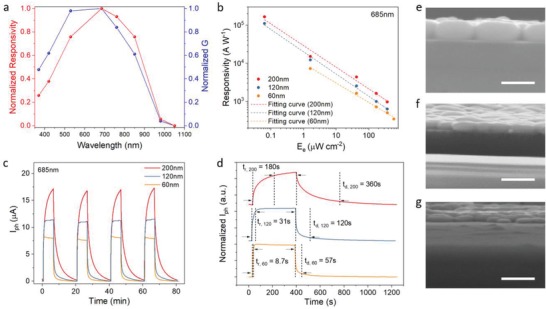
a) Normalized spectral responsivity and gain of the perovskite PD. b) Responsivity versus light intensity for perovskite with different thickness (the dotted lines are fitting curves with a formula of $R\;\propto \;E_{e}^{\beta -1}$). c) Transient response under four on–off illumination cycles for the PDs with various perovskite thickness. d) Normalized photocurrent as a function of time for perovskite PDs with different thickness under one on–off illumination cycle. e–g) Cross‐sectional view of perovskite with thickness of 200 nm (e), 120 nm (f), and 60 nm (g) on SiO_2_/Si. Scale bars represent 300 nm.

The effect of perovskite thickness on device performance was then investigated. In addition to the above discussed 120 nm thick perovskite PD, we further prepared and characterized PDs with perovskite thickness of 200 and 60 nm. As shown in Figure 3b, the responsivity of the PDs increases with the increase of perovskite thickness at any illumination intensity. Figure S6 in the Supporting Information provides supplementary measurement results for 200 and 60 nm thick PDs. The transient properties of the three devices were also characterized, as presented in Figure 3c. It is reasonable to find that the photoresponse is higher for a thicker film. More importantly, the response time of the device decreases with the decrease of the film thickness. As shown in Figure 3d, the PD with the thin perovskite film (60 nm) exhibits a rising time of only 8.7 s while the device with the thickest film (200 nm) shows a rising time of 180 s. Therefore, the device with a thinner perovskite layer can show a faster response speed although the responsivity is slightly decreased. Notably, the photocurrents of all the devices increase rapidly during the initial stage of illumination and then increase slowly, as revealed in Figure 3d. To understand this effect, the rising edges of the transient response are fitted with a double exponential rising function with two time constants

(7) $$I_{\mathrm{ph}}\;=\;A_{1}\;\left(1-\exp\left(-\frac{t}{\tau_{1}}\right)\right)+A_{2}\left(1-\exp\left(-\frac{t}{\tau_{2}}\right)\right)$$

where τ_1_ and τ_2_ are fast and slow relaxation time, and *A*_1_ and *A*_2_ are magnitudes for the two rising components. It can be observed in Figure S5 in the Supporting Information that the experimental results can be fitted very well with the function. As listed in Table S3 in the Supporting Information, rising time of both components can be reduced for two orders of magnitude by simply thinning the perovskite film from 200 to 60 nm. The faster response time for a thinner film can be attributed to the higher carrier density in the film. Because FASnI_3_ perovskite has a strong light absorption in the visible region, most of photons will be absorbed within a thickness of tens of nanometers. Therefore, higher density of carriers can be induced in a thinner perovskite layer under the same light intensity, leading to shorter carrier lifetimes. Consequently, the response time of the PD decreases with the decrease of film thickness. On the other hand, quicker carrier recombination in thinner perovskite layers can induce lower responsivity of the devices.

Flexible PDs have attracted significant research interests due to their broad potential applications.^48^ Considering that the FASnI_3_ perovskite film is thin and flexible, FASnI_3_ PDs were prepared on 50 µm thick polyimide (PI) substrates. **Figure** **4**a shows the *I*_ph_ versus *V*_DS_ relationship for the flexible device. The photocurrent of the device on PI is comparable to that of the device on a SiO_2_/Si substrate (see Figure S6d, Supporting Information). The maximum responsivity is 2.8 × 10^3^ A W^−1^, which is similar to the value obtained from the PD on SiO_2_/Si. After the initial measurements, the device was subjected to a bending test, in which it was manually curved to a radius of ≈8 mm for 300 times, as illustrated in Figure 4b. After the test, the optoelectronic properties were characterized again. As displayed in Figure 4c, the responsivity under different conditions degrades very little after the bending test, indicating an excellent bending stability of the device.

**Figure 4:**
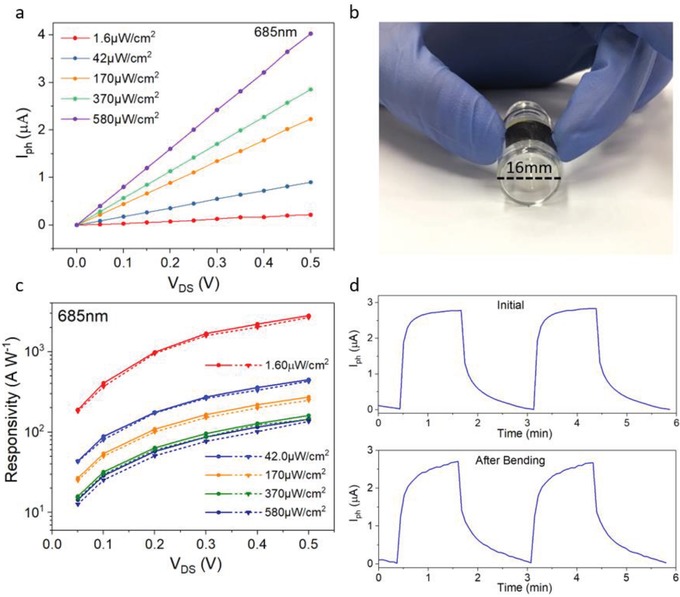
Photoresponse of a 60 nm thick FASnI_3_ PD fabricated on plastic substrate. a) *I*_ph_ versus drain voltage relationship under different light intensities at a wavelength of 685 nm. b) Illustration of the flexible PD subjected to bending test. c) Responsivity as a function of bias voltage for different red light intensity of the flexible PD. Circular and triangular dots are experimental data measured before and after bending for 300 times. d) Temporal response under two on–off illumination cycles, where top and bottom graphs are for performance measured before and after the mechanical testing, respectively.

In summary, PDs based on a single layer of lead‐free perovskite FASnI_3_ demonstrate ultrahigh responsivity and gain that has not been achieved in other perovskite photodetectors with the same device structure. The PD has a broadband photoresponse with the maximum responsivity over 10^5^ A W^−1^. The high performance of the perovskite PD can be attributed to the internal gain of the perovskite material caused by high hole mobility, p‐doping nature, and the excellent optoelectronic property of the material. Faster response speed can be achieved in a device with thinner perovskite layer. Moreover, this type of PD can be prepared on a flexible substrate and demonstrates both high sensitivity and robust bending stability. This work indicates the great potential of lead‐free FASnI_3_ perovskite in the application of low‐cost, sensitive, broadband, and flexible PDs.

## Experimental Section

*Fabrication of Prepatterned Substrate*: SiO_2_ (300 nm)/Si or PI substrates were first cleaned in an ultrasonication bath with deionized water, acetone, and IPA, sequentially, and then dried by blowing the substrate with nitrogen gas. After that, Cr/Au electrodes, with 10 nm of Cr and 100 nm of Au, were patterned by photolithography and thermal evaporation. The channel length (*L*) and width (*W*) were 6 and 800 µm, respectively.

*Formation of FASnI_3_ Film*: 1 m formamidinium iodide (FAI), 1 m tin(II) iodide (SnI_2_) (99.99%), 0.1 m tin(II) chloride (SnCl_2_), 0.1 m dimethyl sulfoxide (DMSO), and 0.015 m hydroquinone sulfonic acid were mixed in a certain amount of *N*,*N*‐dimethylformamide (DMF). The thickness of the perovskite film can be controlled by changing the amount of DMF. Then, the solution was magnetically stirred at room temperature for 6 h in the glove box to obtain the precursor solution for FASnI_3_. After that, the precursor solution was spin‐coated to form a film on SiO_2_/Si. The solution was spun for 30 s at 5000 rpm. 100 µL of chlorobenzene was dropped onto the substrate after spin‐coating for 10 s. Finally, the device was annealed on a hotplate at 70 °C for 5 min.

*Characterization of FASnI_3_*: The structural information of the perovskite film was characterized by X‐ray diffraction, Rigaku SmartLab X‐Ray diffractometer. The perovskite film morphology on SiO_2_/Si was observed under JEOL JSM 6335F scanning electron microscopy. The absorbance of the FASnI_3_ film was recorded with Hitachi UH5300 spectrophotometer.

*Hall Mobility Measurement*: A perovskite film was first spin‐coated on glass substrate following the steps mentioned above. Then, 100 nm of gold electrodes were deposited on the four corners of the film by thermal evaporation. After that, the Hall mobility and carrier concentration were obtained by a van der Pauw Hall measurement configuration using a Hall measurement system (HMS‐3000, Ecopia).

*Electrical, Optoelectrical Measurements for the Devices*: The performance of the devices was measured using a semiconductor parameter analyzer (Keithley 4200) under light illumination at various intensities and wavelengths in a N_2_ gas‐filled glove box. The light sources for this work were LEDs with the wavelengths of 370, 420, 530, 685, 760, 850, 980, and 1050 nm. When measuring the spectral responsivity and EQE, the intensity at all measured wavelengths was set to be about 500 µW cm^−2^.

*Bending Stability Assessment for Flexible Device*: The performance was first characterized using the above equipment for a device assembled on a flexible substrate. Then, it was curved against a little glass bottle with radius of 8 mm for 300 times. Finally, the device performance was measured again. All measurements and bending test were conducted in the glove box to make sure the degradation of the device was only induced by bending tests.

## Conflict of Interest

The authors declare no conflict of interest.

## Supporting information

Supplementary
