# Supplementary material for: Sn‐Based Perovskite for Highly Sensitive Photodetectors
Source: Adv Sci (Weinh). 2019 Jul 13;6(17):1900751. doi: 10.1002/advs.201900751 (PMC6724360; doi:10.1002/advs.201900751)
Supplement: Supplementary file 1 — Supplementary [file ADVS-6-1900751-s001.pdf]

Copyright WILEY-VCH Verlag GmbH & Co. KGaA, 69469 Weinheim, Germany, 2019.

Supporting Information

for *Adv. Sci.,* DOI: 10.1002/advs.201900751

Sn-Based Perovskite for Highly Sensitive Photodetectors

*Chun-Ki Liu, Qidong Tai, Naixiang Wang, Guanqi Tang, Hok-Leung Loi, and Feng Yan**

Copyright WILEY-VCH Verlag GmbH & Co. KGaA, 69469 Weinheim, Germany, 2019.

Supporting Information

Sn-based perovskite for highly sensitive photodetectors

*Chun-Ki Liu, Qidong Tai, Naixiang Wang, Guanqi Tang, H.-L. Loi and Feng Yan**

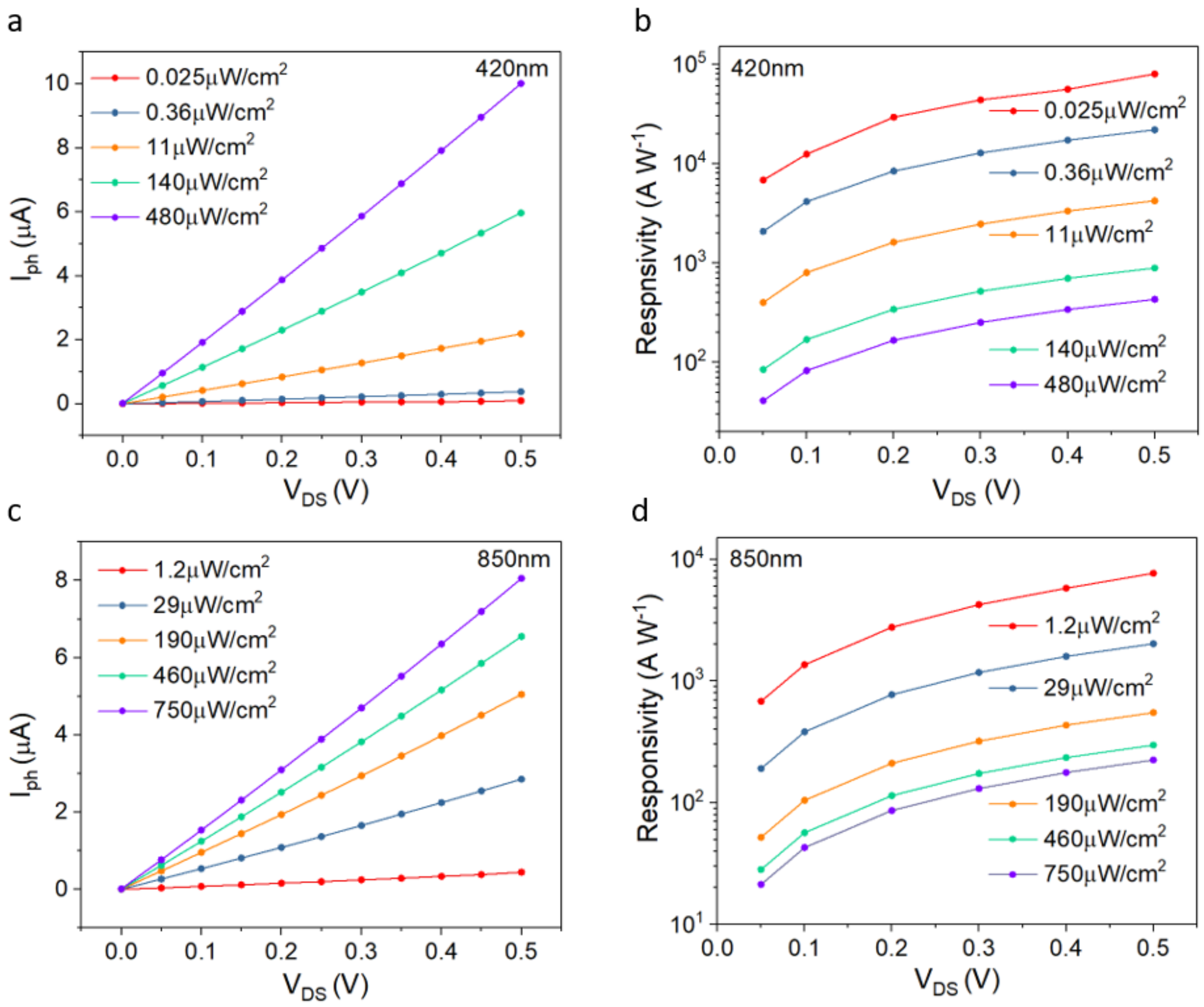

Figure S1. Supplementary device performance for 120 nm-thick perovskite PD. (a) Photocurrent as a function of drain voltage for a $FASnI_3$ device under illumination of light with 420 nm wavelength at different intensities. (b) Responsivity vs. drain voltage relationship under different intensities of light with 685 nm wavelength. (c) Photocurrent as a function of drain voltage for a $FASnI_3$ device under illumination of light with 850 nm wavelength at different intensities. (d) Responsivity vs. drain voltage relationship under different intensities of light with 850 nm wavelength. Solid dots are experimental data.

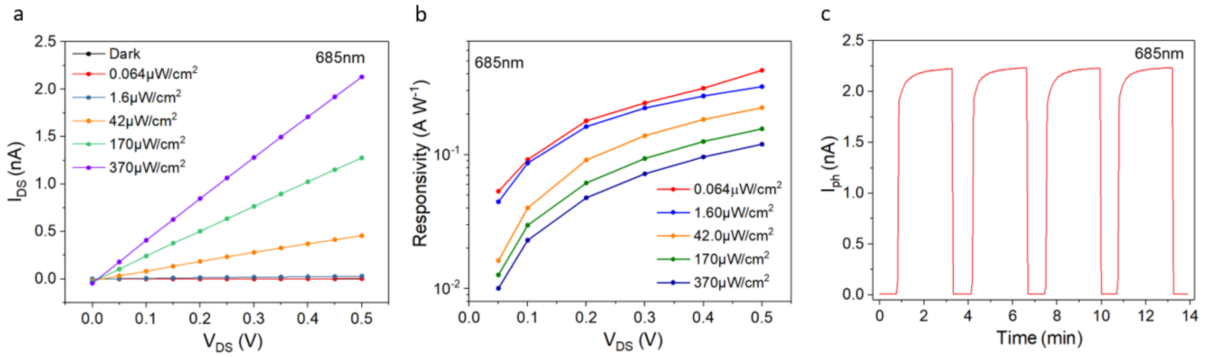

Figure S2. Device performance for a 300 nm-thick $MAPbI_3$ PD. (a) I-V curves in dark and under illumination for the device. (b) Responsivity vs. drain voltage relationship under different intensities of light. (c) Transient response under four on-off illumination cycles for the PD.

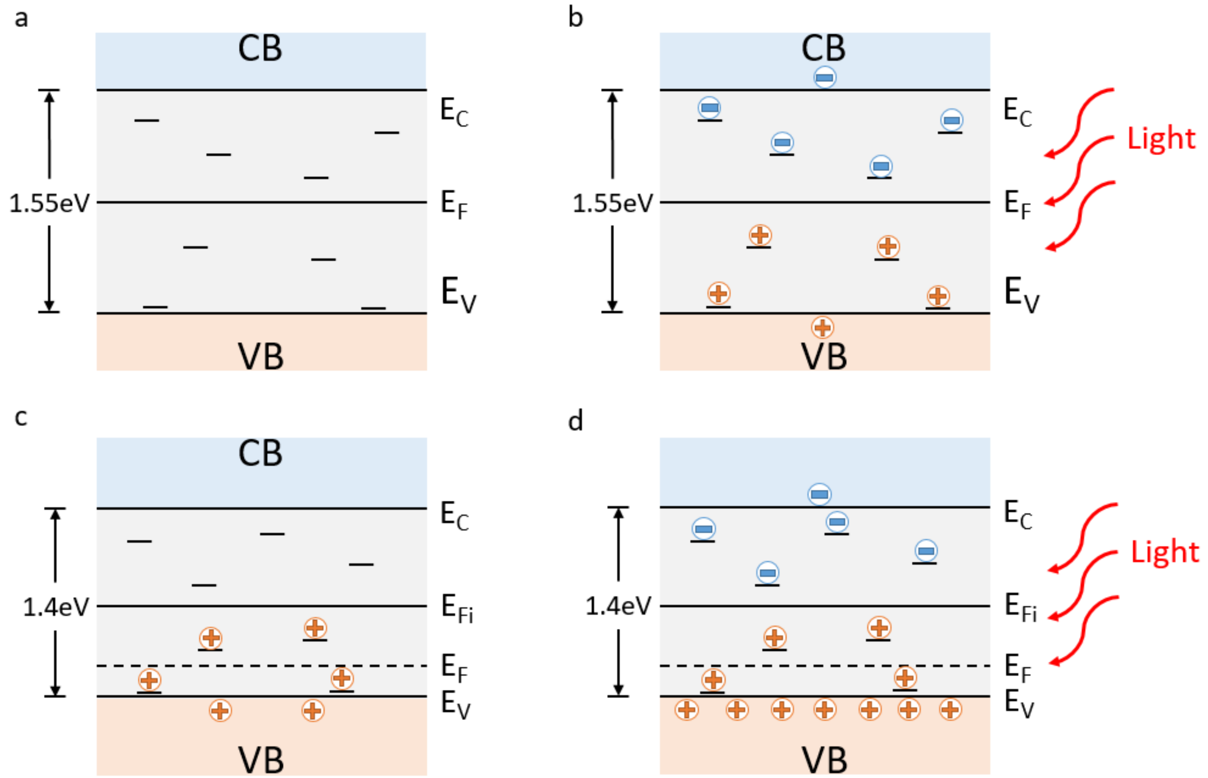

Figure S3. Schematic band diagrams illustrating the charge trapping and transporting mechanisms in $MAPbI_3$ and $FASnI_3$. (a) and (b) are the band diagrams of $MAPbI_3$ without and with light illumination, respectively. (c) and (d) are the band diagrams of $FASnI_3$ without and with light illumination, respectively. In $MAPbI_3$, most of the photo-generated electrons and holes are trapped inside the trap states within the band gap. On the contrary, in $FASnI_3$, only photo-generated electrons are trapped within the band gap, and most of the photo-generated holes are free to move in valance band.

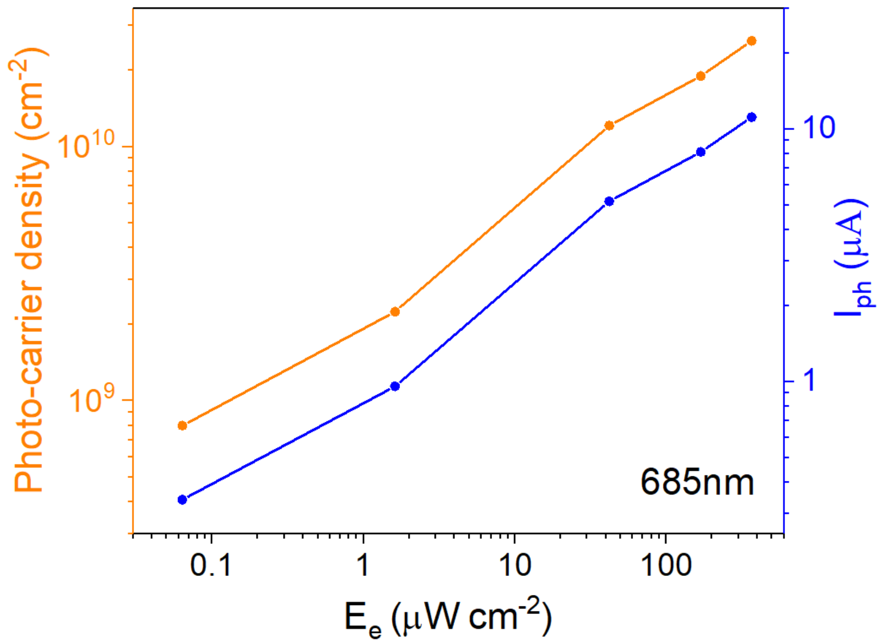

Figure S4. Photo-carrier density and photocurrent as a function of light intensity under 685 nm light illumination for the 120 nm-thick perovskite PD.

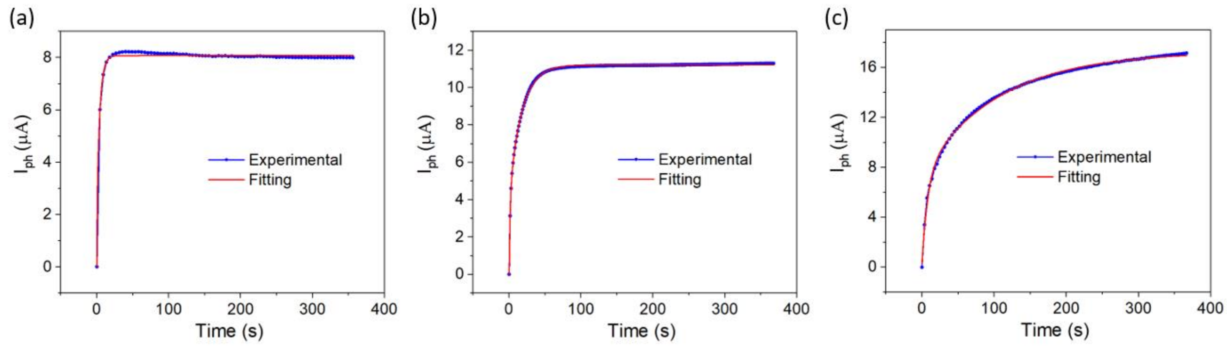

Figure S5. Enlarged Views of the rising edge of the temporal response under illumination with fitting curves for the (a) 60 nm-thick perovskite, (b) 120 nm-thick perovskite and (c) 200 nm-thick perovskite PDs.

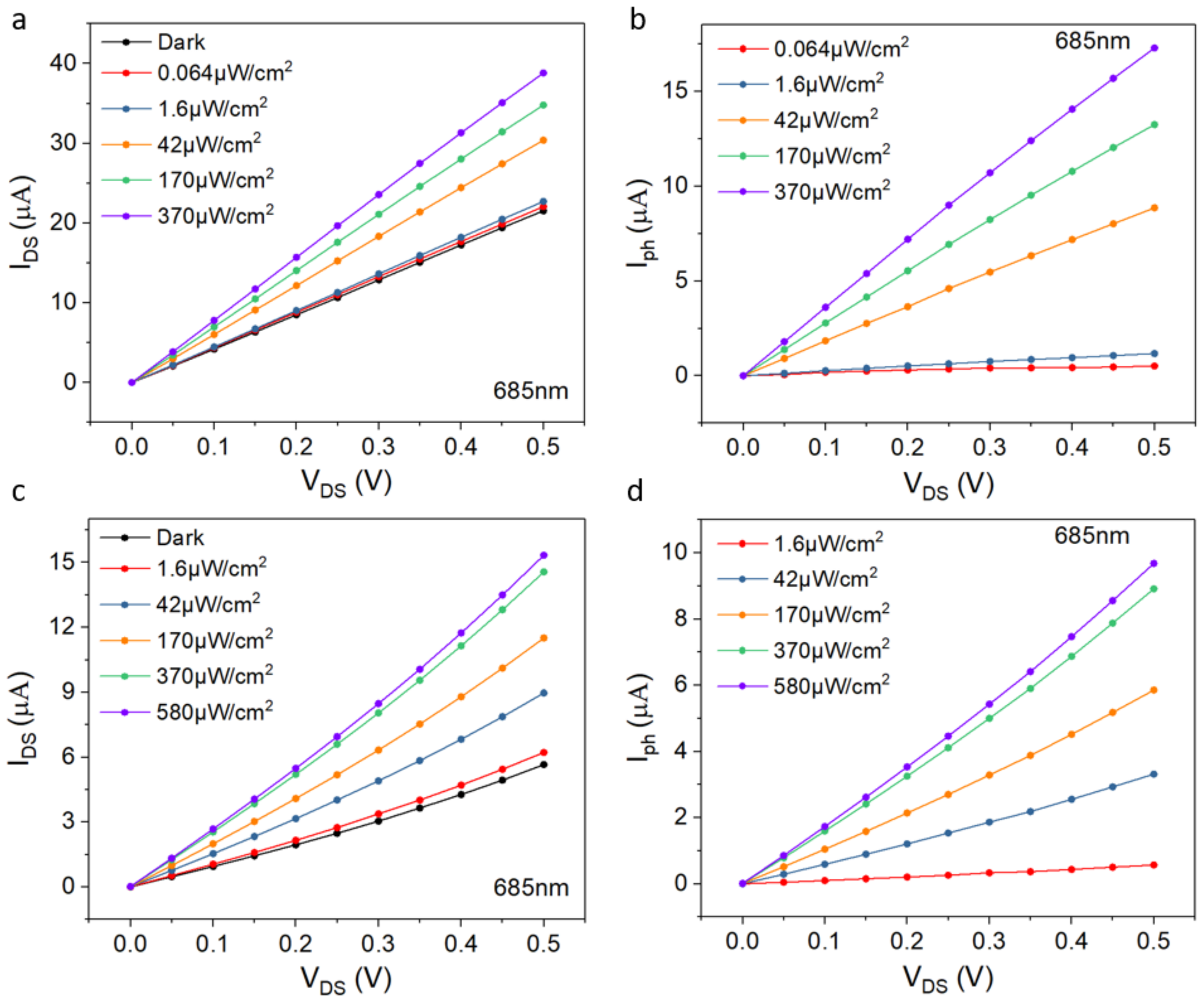

Figure S6. Supplementary device performance for PDs with different $FASnI_3$ film thicknesses. (a) I-V curves in dark and under 685 nm illumination at different intensities and (b) Photocurrent as a function of drain voltage under 685 nm illumination at different intensities for the 200 nm-thick perovskite device. (c) I-V curves in dark and under 685 nm illumination at different intensities and (d) Photocurrent as a function of drain voltage under 685 nm illumination at different intensities for the 60 nm-thick perovskite device.

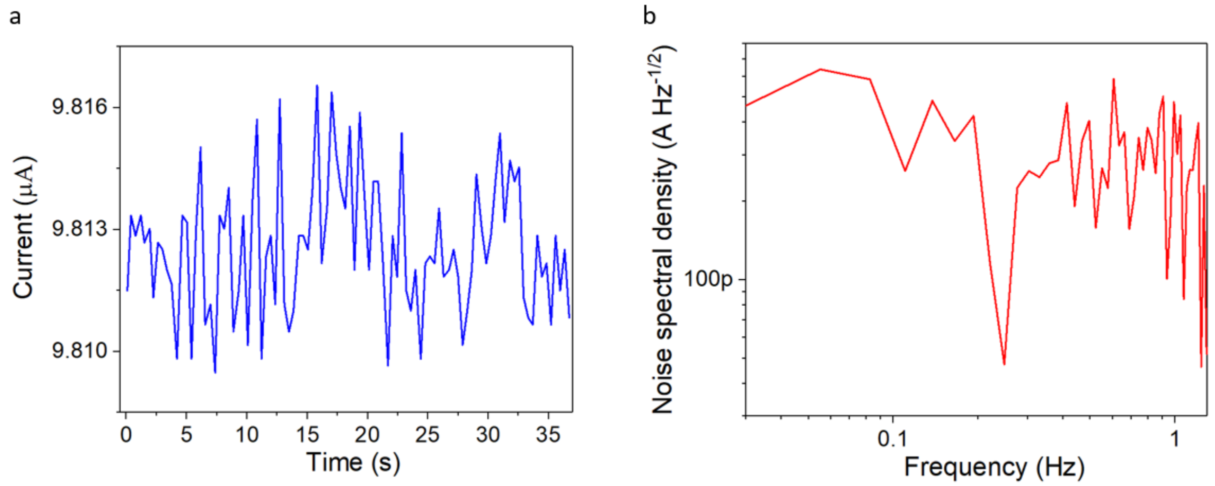

Figure S7. (a)The dark current of the 120 nm-thick $FASnI_3$ PD with V_{DS} = 0.5V. (b) Analysis of noise spectral density of the PD obtained from the dark current shown in (a).

Table S1. Mobility and carrier concentration estimated by Hall effect measurement.

Sample	Mobility (cm^2 V^{-1} s^{-1})	Carrier concentration (10^{16} cm^{-3})
#1	37	0.27
#2	7	13
#3	28	0.35
#4	9	8.4
#5	15	0.85
Mean ± SD	19 ± 13	4.6 ± 4.3

Table S2. Comparasion of the $FASnI_3$ PDs in this work and previously reported PDs with similar structure

Material(s) and structure	Measuring conditions and device geometry	R (A/W)	Gain	Rise time/decay time (s)	Reference
$MAPbI_3$ thin film	V_{DS} = -30 V, λ = broadband, L = 50 µm, W = 1000 µm, V_G = -40V	320	~10-10^2	6.5 x 10^{-6}/5 x 10^{-6}	[13]
$MAPbI_3$ thin film	V_{DS} = 3 V, λ = 365 nm, L = 15 µm, W = 1 cm	3.49	11.9	<0.2/<0.2	[19]
$CsBi_3I_{10}$ thin film	V_{DS} = 1 V, λ = 650 nm, active area = 6 × 10^{-8} m^2	21.8	41.3	0.33 x 10^{-3}/0.38 x 10^{-3}	[28]
$(PEA)_2SnI_4$ thin film	V_{DS} = 5 V, λ = 470 nm, interdigitated electrodes with width and spacing of 10 µm	16	---	0.63/3.6	[29]
$FASnI_3$ thin film (200 nm)	V_{DS} = 0.5 V, λ = 685 nm, L = 6 µm, W = 1000 µm	1.7 × 10^5	3.1 × 10^5	180/360	This work
$FASnI_3$ thin film (160 nm)	V_{DS} = 0.5 V, λ = 685 nm, L = 6 µm, W = 1000 µm	1.1 × 10^5	2 × 10^5	31/120	This work
$FASnI_3$ thin film (60 nm)	V_{DS} = 0.5 V, λ = 685 nm, L = 6 µm, W = 1000 µm	7.3 × 10^3	1.3 × 10^4	8.7/57	This work

Table S3. Rising time for different photodetectors in this work

Structure	τ_1	τ_2
60 nm perovskite	0.34	4.1
120 nm perovskite	1.3	17
200 nm perovskite	8.1	110
